# NeuralFlux: Estimation of Reaction Fluxes at a Genome‐Scale Level From Time‐Resolved Isotope Labelling Patterns Using Deep Learning

**DOI:** 10.1111/pbi.70470

**Published:** 2025-11-20

**Authors:** Sebastian Huß, Zoran Nikoloski

**Affiliations:** ^1^ Systems Biology and Mathematical Modelling Group Max Planck Institute of Molecular Plant Physiology Potsdam Germany; ^2^ Bioinformatics Department, Institute of Biochemistry and Biology University of Potsdam Potsdam Germany

**Keywords:** flux estimation, isotopically nonstationary, metabolic flux analysis, neural networks, stable isotope labelling

Fluxes of intracellular metabolic reactions are an integrated output of multiple cellular processes, from transcription to post‐translational modifications that shape cell physiology (Koley et al. 2024) and can be used to raise targets for improving different metabolic traits (Treves et al. 2022). Intracellular metabolic fluxes are not measured, but are estimated using approaches from Metabolic Flux Analysis (MFA) (Wiechert 2001) that integrate data on (time‐resolved) label enrichment of metabolites, obtained from stable isotope labelling experiments, into a mathematical model of a reaction network with underlying atom transitions. Yet, their estimation at genome scale from time‐resolved isotope labelling data remains unresolved. We present NeuralFlux, a deep learning–based approach that enables the systematic exploration of the design space of stable isotope labelling experiments supporting the planning of informative experiments for genome‐scale flux estimation.

The label enrichments for individual metabolites in a given reaction network can be calculated using a system of coupled ordinary differential equations (ODEs) that describe the change in labelling of a metabolite's pool as a function of steady‐state reaction fluxes, steady‐state (compartmentalised) metabolite concentrations and label enrichments. The inverse problem is that of estimating a steady‐state flux distribution and (compartmentalised) metabolite concentrations for which the simulated label enrichments, using the system of ODEs, minimise a distance measure to the measured label enrichments. Elementary metabolic units (EMUs) (Antoniewicz et al. 2007) reduce the number of variables in simulating label enrichment, and have facilitated genome‐scale flux estimation with data from isotopic stationary states.

By contrast, in experiments with nutrients for which the input labelled atoms cannot be differentiated (e.g., carbon in CO_2_ or nitrogen in NO_3_^−^), data from an isotopic stationary state are not informative as all atoms will be labelled; therefore, in this case, time‐resolved labelling patterns from the transient phase must be used for flux estimation. This has resulted in the development of Isotopically non‐stationary MFA (INST‐MFA) approaches, particularly in photosynthetic organisms (Koley et al. 2024). Despite these advances, flux estimation at a genome scale with INST‐MFA approaches remains challenging (Koley et al. 2024), since existing solutions are applicable to networks of a few reactions (e.g., INCA; Young 2014) or are computationally demanding, affecting reproducibility (Gopalakrishnan et al. 2018).

Given a reaction network together with a set of sampled steady‐state flux distributions and (compartmentalised) metabolite concentrations, our approach, termed NeuralFlux, makes use of the corresponding system of ODEs to simulate label enrichments, in a form of mass isotopomer distributions (MIDs), for the EMUs at time points corresponding to experimental measurements (Figure 1c). NeuralFlux then uses a steady‐state flux distribution along with (compartmentalised) metabolite concentrations as input to a fully connected neural network with three hidden layers of 216, 36 and 6 nodes, respectively, trained to predict the MID of one EMU at a given time point. Given that the average standard deviation of the simulated MIDs is lower than the standard deviation of typical MID measurements (Figure 1g), these neural networks are used instead of numerically solving the system of ODEs in flux estimation. The solution space of NeuralFlux is defined by the sampled values used in training the neural networks, and their bounds require usage of information from literature. This approach leads to a reduction in computational effort by two to three orders of magnitude (Figure 1h).

**FIGURE 1:**
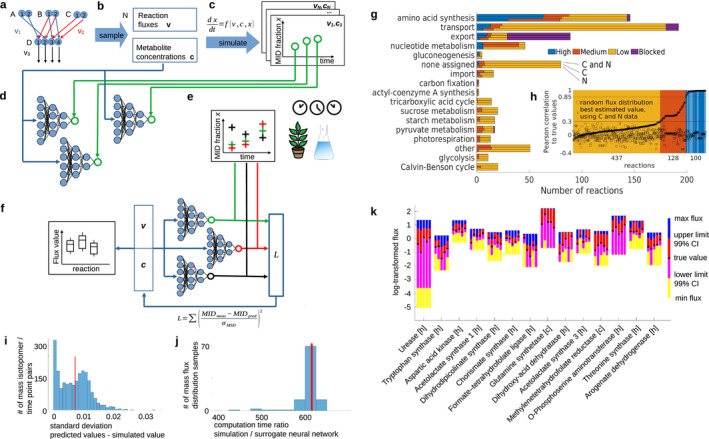
The workflow of NeuralFlux, evaluation of estimated fluxes, and their confidence intervals with labelling data from amino acids in a large‐scale model of *A. thaliana* . (a) A reaction network toy model with metabolites A–D, three reactions including atom transitions denoted with arrows. (b) *N* samples of steady‐state flux distributions and metabolite concentration distributions. (c) Time‐resolved mass isotopomer distributions (MIDs) generated by numerically solving the system of ordinary differential equations. (d) Training of neural networks that predict MIDs using the generated data samples with the corresponding simulated MIDs. (e) Experimental measurements of MIDs at specific time points. (f) Estimation of metabolite concentrations and steady‐state fluxes which minimise the reduced chi‐square statistic L between predicted and measured MIDs. (g) Standard deviation between simulated MID values and MID values inferred by the surrogate neural networks for most MID/time‐point pairs; median is marked in red. (h) Ratio of computational time needed to simulate MID values and inferring the values by surrogate neural networks for 100 runs. Median is marked in red. (i) The × symbol (square) in black denotes the Pearson correlation for the estimated values of a reaction (for a randomly chosen flux sample) and the true simulated values for simulated labelling measurements of 34 metabolites. Reactions shown in (j) are marked with a white line. We identified three groups of reactions based on their Pearson correlation: (*i*) 437 have < 0.3 (yellow); (*ii*) 128 are between 0.3 and 0.85 (orange); (*iii*) 100 reactions have > 0.85 (blue). (j) The calculated 99% confidence intervals vary between reactions within a flux distribution, but also for a reaction between flux distributions (14 reactions in 5 flux distributions shown). Reaction locations are cytosol [c] and chloroplast [h].

NeuralFlux provides statistically sound flux estimates, as it relies on the Trust‐Region‐Reflective Least‐Squares approach (Moré and Sorensen 1983) that considers the number of measurements and degrees of freedom (in the present study, 2040 measurements and 1187 degrees of freedom) in optimising the reduced chi‐square statistic. The calculation of the 99% confidence interval in NeuralFlux is performed for the estimated flux values of each reaction individually, following an established approach from MFA (Antoniewicz et al. 2006).

To demonstrate the advances made by NeuralFlux, we investigated a metabolic model of primary metabolism of *Arabidopsis thaliana* , AraCore v2.1 (Wendering et al. 2025), including 585 compartmentalised reactions and 415 compartmentalised metabolites, located in four compartments, that is, cytoplasm, chloroplast, mitochondrion and peroxisome. In our proof‐of‐concept study, we considered the 20 amino acids and 14 further metabolites as measured in the evaluation scenarios for nitrogen and carbon labelling. Detailed descriptions of NeuralFlux and the proof‐of‐concept are provided in the Supporting Information.

To quantify how well a reaction flux can be estimated from the set of these measured metabolites, we first used 100 randomly sampled steady‐state flux distributions and estimated the flux values from the simulated enrichment values, using NeuralFlux with compartmentalised metabolite concentrations as input. We employed the Pearson correlation coefficient between the estimated and true flux values as a performance measure. We found that 100 reactions show Pearson correlations > 0.85 and significantly different from correlations to random fluxes (Figure 1i), denoting them as well estimated. This finding indicates that already with these few simulated measurements of metabolites and time points, NeuralFlux provides flux estimates that match the trend of simulated, true fluxes for ~one‐sixth (100/585) of reactions; this result also demonstrates that additional labelling data are needed to differentiate estimates for the remaining reactions.

To better characterise the flux estimates, we evaluated the 99% confidence intervals for 14 of the well estimated reactions in five flux distributions. We found that the 99% confidence intervals differed between reactions in a single flux distribution (e.g., chorismate synthase and formate‐tetrahydrofolate ligase), but also between flux distributions for a single reaction (e.g., dihydroxy‐acid dehydrogenase) (Figure 1j). These findings indicate that obtaining precise flux estimates in plant metabolism at the genome‐scale level would require either greater precision and a larger number of measured MIDs at well‐chosen time points or imposing additional constraints known to shape the functionality of the network, including the ability to obtain compartment‐specific labelled data. In conclusion, NeuralFlux provides a feasible approach to rapidly test such scenarios due to the low computational demands.

## Conflicts of Interest

The authors declare no conflicts of interest.

## Supporting information


**Data S1:** pbi70470‐sup‐0001‐Supinfo.pdf.

## Data Availability

The data that support the findings of this study are openly available in Zenodo at https://doi.org/10.5281/zenodo.17426172. NeuralFlux and all data used in its evaluation are available under the Apache Licence Version 2.0 at https://github.com/sebahu/NeuralFlux.
