# Supplementary material for: NeuralFlux: Estimation of Reaction Fluxes at a Genome‐Scale Level From Time‐Resolved Isotope Labelling Patterns Using Deep Learning
Source: Plant Biotechnol J. 2025 Nov 20;24(4):2076–8. doi: 10.1111/pbi.70470 (PMC13140539; doi:10.1111/pbi.70470)
Supplement: Supplementary file 1 — Data S1: pbi70470‐sup‐0001‐Supinfo.pdf. [file PBI-24-2076-s001.pdf]

NeuralFlux: Estimation of reaction fluxes at a genome-scale level from time-resolved isotope labeling patterns using deep learning

Sebastian Huß[1,2] and Zoran Nikoloski[1,2*]

[1]Systems Biology and Mathematical Modelling Group, Max Planck Institute of Molecular Plant Physiology, Potsdam, Germany.
[2]Bioinformatics Department, Institute of Biochemistry and Biology, University of Potsdam, Potsdam, Germany.
*corresponding author: nikoloski@mpimp-golm.mpg.de

Supplemental Material

Methods

Overview

NeuralFlux estimates fluxes together with their confidence intervals provided data from stable isotope labeling experiments, where isotope labeling is still in a transient state. A mathematical model for the network of biochemical reactions and underlying atom transitions are also used as input. This model is used to specify a system of coupled ordinary differential equations (ODEs) that describes the change of mass isotopomer distributions (MIDs) for all relevant elementary metabolic units (EMUs[1]). To simulate the evolution of MIDs, this system of ODEs requires a steady-state flux distribution and a set of compartmentalized metabolite concentrations as parameters.

In NeuralFlux, the system of ODEs is solved numerically for a large number of sampled steady-state flux distributions and metabolite concentrations, used as input data sets, the resulting simulated MIDs for the EMUs at selected time points of the system are stored. The collected data are then used to train neural networks, where each neural network has flux values and compartmentalized metabolite concentrations as input (all neural networks have the same form of input) and predicts the fraction of one mass isotopomer of one EMU at a given time point. The flux estimation for the measured EMU MIDs of one labeling experiment is then a parameter fitting problem, with the flux distribution and the compartmentalized metabolite concentrations as parameters. The squared distance between the MID values predicted using the neural networks and the measured MID values is minimized using the Trust-Region-Reflective Least-Squares approach[2], necessitating that the number of fitted data points is greater than the number of free fluxes and compartmentalized metabolites, to guarantee a positive value for the degrees of freedom.

Prerequisites and Preparations

The main prerequisite to applying NeuralFlux to experimental data is a metabolic model for the studied system, that: (*i*) models the metabolism of the organism from the import of the labeled nutrient to biomass production and (*ii*) includes all pathways that lead to the measured metabolites. The model must be feasible, *i.e.*, it must be possible to find flux distributions that represent a metabolic steady state in the model and result in biomass production (growth).

As a proof of concept, we made use of a large-scale metabolic model of *Arabidopsis thaliana*, AraCore v2.1[3]. The model contains the nitrogen and carbon primary metabolism of *A. thaliana*, including 585 compartmentalized reactions located in four compartments, *i.e.*, cytoplasm, chloroplast, mitochondrion, and peroxisome. These reactions include transport reactions and three biomass reactions, modeling growth under different environmental conditions, and 156 reversible reactions. The model contains 415 compartmentalized metabolites, comprising of 240 unique metabolites, of which 88 are present in more than one compartment. The model was transformed by splitting reversible reactions into forward and reversed reactions, thus having only irreversible reactions with a non-negative lower bound for their flux. The original AraCore v2.1 model and the transformation code is part of the GitHub repository (https://github.com/sebahu/NeuralFlux/).

In addition, for all reactions leading to the measured metabolites, there must exist atom mappings for the labeled element. If (some of) those mappings do not exist, they can be retrieved from online databases such as MetaCyc[4] or generated with tools like RDT[5]. To enable the combined usage of all these atom mappings, they need to have unified atom identifiers, *i.e.*, one specific atom of a metabolite must have the same identifier in the mappings of all reactions in which the metabolite participates. The mappings used in this work are based on the results of a previous work[6]. These existing mappings were adapted to changes in the used newer version of the metabolic model, resulting in 1603 mappings for individual nitrogen atoms in 339 reactions and 6421 mappings for individual carbon atoms in 457 reactions. The mappings are stored in text files for carbon and nitrogen respectively, with one line containing the mapping of one substrate/product atom pair in one reaction. These updated mappings are part of the GitHub repository (https://github.com/sebahu/NeuralFlux/).

Some aspects of the experimental setup need to be captured at this stage of the approach, namely the influx of the labeled nutrient and the set of potentially measured metabolites. To model the influx of labeled nutrients, the EMU mass isotopomer (MI) and the specific influx reaction need to be specified. For the carbon labeling example, the reaction is ´Im_CO2´, CO_2 import and the EMU MI is ´CO2[c]:C#1.1´, carbon dioxide molecules in the cytoplasm containing one labeled carbon atom. For the nitrogen example, the reactions are ´Im_NO3´, nitrate import, and ´Im_NH4´, ammonium import. The corresponding EMU MIs are ´NO3[c]:N#1.1´ and ´NH4[c]:N#1.1´, nitrate and ammonium ions in the cytoplasm containing one labeled nitrogen atom. For the metabolites that are planned to be measured in experiments, the EMUs of their actually measured fragments must be specified. In general, this will be the EMU that contains all atoms of the labeled element. For metabolites, where the measured fragment does not contain all the atoms of the labeled element, the specified EMU must contain exactly the atoms in the measured fragment.

Lastly, if information about the most likely absolute metabolite concentration is available from the literature or measurements for a set of metabolites, this should be included in the setup, as it is used in the sampling of metabolic states.

Sampling of metabolic states

The sampling of flux distributions and metabolite concentrations defines the solution space of the approach. Ideally, it covers all feasible flux distributions and metabolite concentrations of the experimental setup. It may cover a larger space, but if sufficient information from the experiment allows a reduction of the sampling space, an improved precision or reduced execution time (by using fewer samples) is to be expected. The resulting samples must be statistically analyzed to determine whether they actually cover the intended space of metabolic states.

In the presented examples, the aforementioned modified AraCore v2.1 model was sampled using Coordinated Hit and Run with Rounding (CHRR[7]) from the COBRA toolbox[8]. To limit the sampling space in a meaningful way while still allowing a wide range for all fluxes, several constraints were added to the model for sampling. First, the maximum biomass production was calculated. The minimum sum of all fluxes of the model was then determined, while biomass production still reaches the optimum, using parsimonious flux balance analysis (pFBA)[9]. For the sampling, the total sum of fluxes was then limited to twice the minimum identified from pFBA. To increase the range of flux distributions, the sampling was done in six batches of 100,000 samples with the lower bound of biomass production set to 0.3, 0.5, 0.65, 0.8, 0.9 and 0.95 of the optimum for the nitrogen labeling example, and twelve batches of 50000 samples with the lower bound of biomass production set to 0.3, 0.4, 0.5, 0.575, 0.65, 0.725, 0.8, 0.85, 0.9, 0.925, 0.95 and 0.975 for the carbon labeling example. Furthermore, amino acid export reactions were limited, so that the amount of exported amino acids is at most of comparable size to the amount used in the biomass production reaction.

In total, in both examples 600,000 flux distribution samples were created. If a reaction can be measured directly in the labeling experiment, this can be used to reduce the complexity without loss of variance by normalizing the samples according to this reaction. In the examples, the ammonium and nitrate import reactions were assumed to be measurable, and the samples were normalized according to their sum in relation to the maximum of this sum over all samples.

The metabolite concentrations are sampled independently of the fluxes. The total concentration of a metabolite in all compartments is chosen from a random logarithmically uniform distribution from 0.01 to 0.1 umol/gDW, while metabolites with a concentration value from literature or actual measurements are chosen from a random logarithmically uniform distribution from 0.2 to 2-fold of this value. The relative metabolite concentrations in mitochondrion, plastid, and peroxisome are taken from uniform distributions in the ranges of 0.05-0.15, 0.15-0.4 and 0.01-0.05 when the metabolite is contained in that compartment, the rest is allocated to the cytoplasm. The total concentration of a metabolite is then divided between compartments according to relative concentrations. The total number of compartmentalized metabolite concentrations created is equal to the number of flux distributions (*i.e.*, 600,000).

Simulation of labeling patterns

The simulation of metabolite labeling is implemented using elementary metabolic units (EMUs[1]). EMUs represent parts of a molecule that remain intact in a chemical reaction. For instance, when ATP reacts to ADP and phosphate, the ADP molecule is an EMU, as is the according part of the ATP molecule. For the tracking of a labeled element, only the atoms of this element are relevant. In preparation of the simulation, the measured EMUs have been specified. To successfully simulate the labeling of the measured EMUs, all intermediate EMUs on the pathways from the nutrients to the measured metabolites must be simulated. Existing approaches identify these needed EMUs in a simple Breadth-First Search (BFS), which takes all reactions that produce the metabolites of the needed EMUs and calculates the substrate EMUs from the atom mappings. Those EMUs are added to the set of needed EMUs until no new EMUs are found. A special case are EMUs involved in bimolecular reactions in which a product EMU is synthesized from two substrate EMUs. Here, the two substrate EMUs together form one EMU; however, the individual substrate EMUs still need to be simulated.

For every EMU with n atoms of the labeled element, there exist $n+1$ mass isotopomers (MIs). The change of concentration for each MI belonging to an individual EMU can be calculated from the flux values of all reactions with the EMU's metabolite as a product or substrate, in combination with the fractions of the MIs with the same number of labeled atoms of the EMU, and all substrate EMUs. This results in the following ordinary differential equation (ODE):

$$
\begin{aligned}
\frac{dx_{A,m}}{dt} &= \frac{\sum_{i=1}^{n} v_i x_{S_i,m} - x_{A,m}\sum_{j=1}^{o} w_j}{c_A} \\
\text{with} &: \sum_{i=1}^{n} v_i = \sum_{j=1}^{o} w_j \ \text{(steady state)} \\
x_{A,m} &: \text{fraction of EMU } A \text{ with } m \text{ labeled atoms} \\
c_A &: \text{concentration of EMU } A \\
n &: \text{number of reactions with } A \text{ as product} \\
v_i &: \text{flux of the } i\text{-th reaction with } A \text{ as product} \\
x_{S_{i,m}} &: \text{fraction of EMU } S_i \text{ (the direct substrate of } A \text{ in the} \\
&\quad i\text{-th reaction with } A \text{ as product) with m labeled atoms} \\
o &: \text{number of reactions with } A \text{ as substrate} \\
w_j &: \text{flux of the } j\text{-th reaction with } A \text{ as substrate}
\end{aligned}
\tag{1}
$$

The ODEs can be solved with any numerical algorithm (*e.g.* Runge-Kutta method, Midpoint method, Bulirsch-Stoer algorithm). In this work, a simple Euler method with a sufficiently small time step was used to numerically solve the ODEs. This was implemented using a mapping matrix M, with rows and columns representing the EMU MIDs to be simulated. Additional rows represent the MIDs for EMUs from synthesis reactions, which are combined from two substrate EMUs. These EMUs have special MIs that also address the split of the MI and identify how

many labeled atoms in the combined EMU come from each partial EMU. An additional column represents the flux of reactions that are not leading to any of the needed EMUs, *e.g.*, export or biomass reactions. The value in row i and column j denotes the sum of all reaction fluxes for one time step in which EMU-MI i is converted to EMU-MI j. The total out-flux of one EMU MI equals the sum of the corresponding row multiplied with the fraction of the specific MI of all MIs of the EMU. The in-flux into one EMU MI is the sum of the corresponding column, multiplied element-wise by the fractions of the corresponding substrate EMU MIs. For substrate EMUs of synthesis reactions, which combine two EMUs, the fact that one MI for the combined EMU can have multiple combinations with MIs of the individual EMUs is handled accordingly (equation (2)).

$$
\begin{aligned}
x_{(B+C),m} &= \sum_{\substack{n \geq 0 \\ o \geq 0 \\ n+o=m}} x_{B,n} x_{C,o} \\
x_{(B+C),m} &: \text{fraction of EMU } (B+C), \text{ combined from EMUs } B \text{ and } C \\
&\quad \text{in a condensation reaction, with } m \text{ labeled atoms in total} \\
x_{B,n} &: \text{fraction of EMU } B \text{ with } n \text{ labeled atoms} \\
x_{C,o} &: \text{fraction of EMU } C \text{ with } o \text{ labeled atoms}
\end{aligned}
\tag{2}
$$

The mapping matrix, M, is also used to check the consistency of the atom mappings. If M is constructed with a flux distribution guaranteeing metabolic steady state, and the atom mappings are consistent, then the matrix represents a steady state of the EMU MIDs. As a result, the sum of inputs equals the sum of outputs for every EMU, which is not a combination of other EMUs.

The simulated time period and the number of intervals after which intermediate MIDs are stored are both parameters depending on the experimental setup. The number of steps - and thus the step length - for the Euler method is chosen in such a way that the turnover rate of each EMU for this time span is below 0.5. The maximum number of steps in the implementation is 20,000. If an EMU has a turnover rate in one step of more than 0.5, the metabolite concentration of this EMU for the simulation is increased so that the turnover rate is 0.5. Since the turnover of those metabolites is still very high, the increased concentration has only effects on the simulated enrichment that are negligible compared with the precision of the actual measurements.

Analysis of the simulated enrichment data

The simulated enrichment data are used in the next step of NeuralFlux to train neural networks that replace the ODEs for the final flux estimation step. The simulated enrichment data can also be analyzed to improve the experimental setup and reduce the number of neural networks that need to be trained. For instance, the most informative time points for one MID can be determined by statistical analysis. Furthermore, the metabolites to be measured in the actual experiment can be chosen from the potential candidates by the discriminating power they show in the simulated enrichment data. Finally, the set of neural networks can be further reduced if the fractions of one or a few MIs of an EMU already contain most of the information about the other MIs of the EMU. In the nitrogen examples, examined in this work, the fraction of only MI m_0 was already found to be informative and potentially used as the only MI used for each EMU. We note that in Kinetic flux profiling[10] also makes use of only the unlabeled fraction of metabolites. We note that the number of pairs of MIs and time points corresponds to the number of observations for the final optimization step of NeuralFlux. Therefore, to obtain meaningful optimization results, the number of observations must be large enough compared with the number of free fluxes and compartmentalized metabolite concentrations to result in a positive degree of freedom.

Architecture and training of neural networks in NeuralFlux

The main idea of the presented flux estimation approach is the replacement of the actual ODEs with neural networks that are, in turn, used during the actual flux estimation step. We train one neural network per decompartmentalized metabolite MI and time point. While it is possible

to train neural networks that predict all time points of an MI or all MIs at one time point, the limitation to only one output has several advantages: First, the individual training problems are simpler than with multiple outputs. Second, this enables full modularity and scaling, since only the neural networks for the measured time points of the measured metabolite MIs need to be trained, allowing for easy parallelization.

All individual neural networks have the same input nodes, the sampled flux distribution, and relevant compartmentalized metabolite concentrations. The flux distribution is represented by the factors of the a selected basis for the nullspace of the stoichiometric matrix. This re-parameterization ensures that every instance of these parameters represents a flux distribution that leads to a metabolic steady state. The relevant compartmentalized metabolite concentrations are the concentrations of all metabolites which are necessary to calculate the measured MI values, *i.e.*, which occur in the mapping matrix of the enrichment simulation. For the nitrogen labeling simulation of the AraCore model, this amounts to 356 null-space factors or free fluxes and 182 relevant compartmentalized metabolite concentrations. To improve performance, every input parameter to the neural network was standardized in z-score across all 600,000 samples (equation (3)).

$$
\begin{aligned}
Z_i &= \frac{X_i - \mu_i}{\sigma_i} \\
Z_i &: \text{standardized sample values for parameter } i \\
X_i &: \text{sampled values for parameter } i \\
\mu_i &: \text{mean of the sampled values for parameter } i \\
\sigma_i &: \text{standard deviation of the sampled values for parameter } i
\end{aligned}
\tag{3}
$$

As a configuration of the hidden layers, three fully connected layers of size 216, 36, and 6 nodes were chosen, which led to good results. The commonly used standard activation function used in regression neural networks is the rectified linear unit (ReLU) function, yet this results in problems during the optimization for the actual flux estimation. Consequently, a sigmoid activation function was used instead, which is well suited in our setting, since its range of [0,1] corresponds to that of the MID fractions.

Experimental data

The measured experimental data are decompartmentalized metabolite MIDs and absolute concentrations. For NeuralFlux, the MID data are the main inputs, and the absolute concentration is used for additional constraints on the compartmentalized metabolite concentration parameters. NeuralFlux can also be applied without the absolute concentrations; however, this leads to reduced precision. If one or more fluxes were directly measured, *e.g.*, nutrient uptake or growth, and the flux distribution samples used to train the neural networks were normalized according to this reaction, this has also to be performed for the experimental data.

Flux estimation

The flux estimation is performed by fitting the parameters to the neural networks (flux distribution and compartmentalized metabolite concentrations), so that the squared distance of the neural network predictions to the measurements of the experiment is minimized. This was implemented using the MatLab function lsqnonlin, which in turn uses the Trust-Region-Reflective Least Squares method[2] if the degrees of freedom are positive, which needs to be ensured in the setup. To find good starting values for the parameters, the five samples (containing flux distribution and compartmentalized metabolite concentrations) with the smallest sum of squared differences between the observed MID values and the simulated MID values for the samples are determined. The average flux and metabolite concentration values of these samples are then used as a starting value for optimization. The function that is minimized is the reduced chi-squared statistic (equation (4)).

$$
\begin{aligned}
\chi^2_\nu =& \frac{\chi^2}{\nu} \\
\nu =& n - m \qquad \text{degrees of freedom} \\
\chi^2 =& \sum_{i=1}^{n} \frac{(o_i - c_i)^2}{\sigma_i^2} \\
n :& \text{ number of observations} \\
m :& \text{ number of parameters} \\
o_i :& \text{ observed value } i \\
c_i :& \text{ calculated value } i \\
\sigma_i :& \text{ standard deviation for observed value } i
\end{aligned}
\tag{4}
$$

σ_i standard deviation of observations O observations C calculated data

In our evaluation, we set the standard deviation of the observations to a constant value of 0.01, which is in the standard deviation range for this kind of measurements (in a study containing ^{13}C labeling experiments with human liver tissue[11] only standard deviations in replicated measurements of at least 0.03 were accepted.), as well as in the range of the additional error introduced by using values predicted from NNs instead of solving ODEs. With actual experimental data, the standard deviation can be set to the calculated value over replicas of each observation or over groups of observations. The optimization is halted, when χ^2_ν reaches 1, ensuring a statistically acceptable fit without overestimation.

Optionally, the available information on absolute concentrations of decompartmentalized metabolite concentrations can be integrated into optimization by adding a barrier function that adds a large penalty if the sum of compartmentalized concentrations of one metabolite is outside a defined range of the measured decompartmentalized concentration value. The penalty is large enough to prevent reaching a χ^2_ν of 1.

To evaluate the theoretically best estimate of flux values only in one scenario, we used the actual compartmentalized metabolite concentrations used to create the test data, only allowing a small range of values around the correct value.

Calculation of confidence intervals

To calculate confidence intervals for the estimated flux values, we follow an established approach for MFA[12]. The optimization problem is now extended by a barrier function that keeps one individual flux, calculated from the free fluxes, above or below a fixed value. This flux value is then increased and decreased from the fixed value of the optimal case using a barrier function, as for the compartmentalized metabolite concentrations, until the resulting χ^2_ν is sufficiently increased according to the intended confidence level for one variable. In our evaluation, we used 6.67 for a confidence level of 99%.

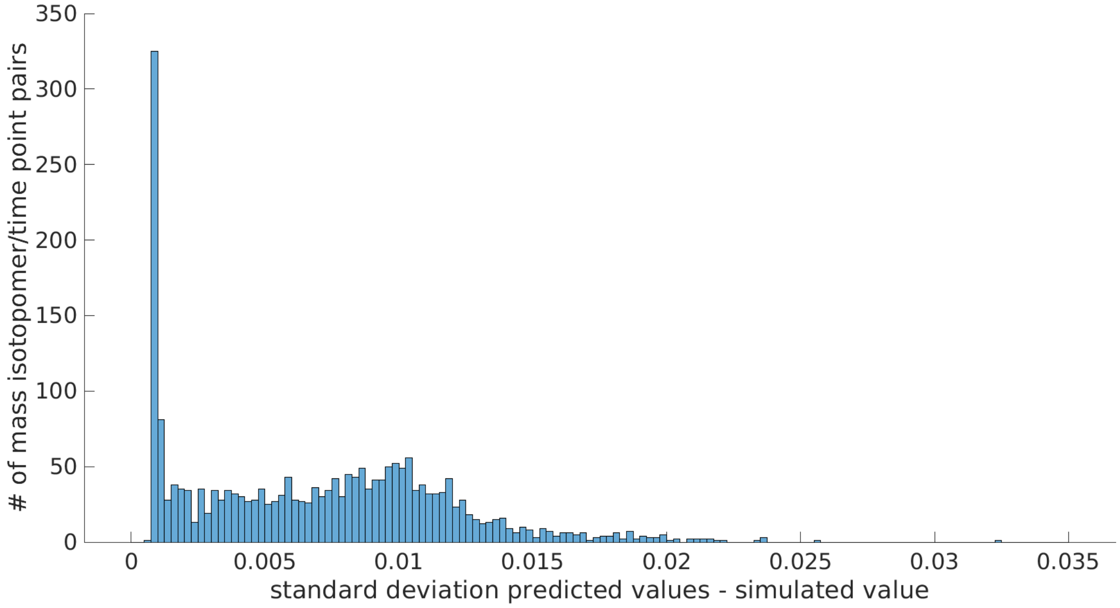

Supplemental fig. 1: Histogram of the standard deviation of the difference between the predicted and simulated values for mass isotopomers. The standard deviation for over mass isotopomers (MIs) at different time points in 12000 samples exhibits an average of 0.007. For the measurement of MIs in labeling experiments, a standard deviation of at least 0.01 is assumed. In comparison, for high-resolution ^{13}C MFA, a standard deviation of at most 0.003 is recommended[13]. Yet, in studies covering ^{13}C labeling experiments, higher standard deviations can be observed, *e.g.* a study on human liver tissue[11] accepted standard deviations of at least 0.03.)

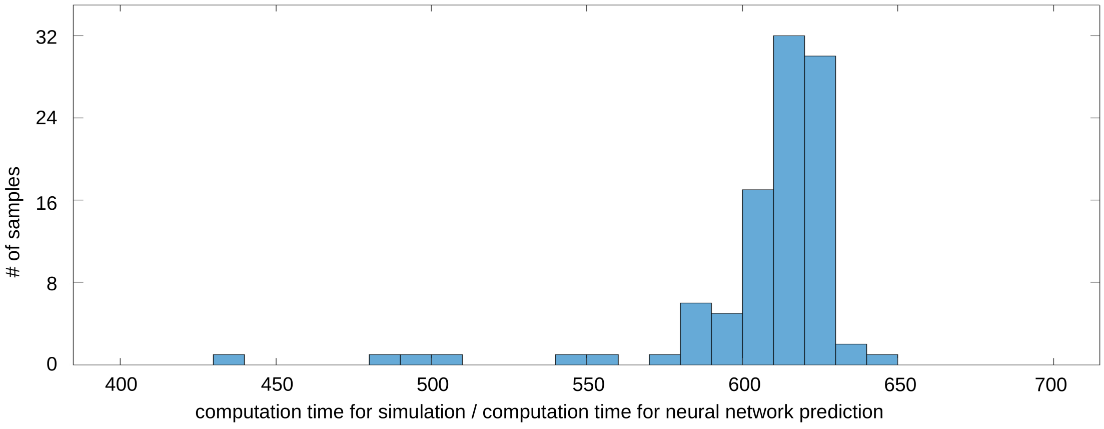

Supplemental fig. 2: Histogram of computational speed-up for predicting values with neural networks instead of ODE simulation. Predicting the fractions of 137 EMU mass isotopomers at 10 time points for one sample of fluxes and metabolite concentrations leads to, on average, 607-fold reduction in computation time compared with simulating the labeling enrichment for the same sampled data. This evaluation was performed on a single core of an AMD EPYC 7542 CPU.

Data availability

The AraCore v2.1 model, atom transition mappings, and metabolite concentrations, obtained from literature, used in the evaluation of NeuralFlux are available at https://github.com/sebahu/NeuralFlux/models.

Code availability

NeuralFlux is available under the Apache License Version 2.0 at https://github.com/sebahu/NeuralFlux.

References

1. Antoniewicz, M. R., Kelleher, J. K. & Stephanopoulos, G. Elementary metabolite units (EMU): a novel framework for modeling isotopic distributions. *Metabolic engineering* **9,** 68–86 (2007).
2. Moré, J. J. & Sorensen, D. C. Computing a trust region step. *SIAM Journal on scientific and statistical computing* **4,** 553–572 (1983).
3. Wendering, P., Andreou, G. M., Laitinen, R. A. & Nikoloski, Z. Metabolic modeling identifies determinants of thermal growth responses in Arabidopsis thaliana. *New Phytologist* (2025).
4. Caspi, R. *et al.* The MetaCyc database of metabolic pathways and enzymes-a 2019 update. *Nucleic acids research* **48,** D445–D453 (2020).
5. Rahman, S. A. *et al.* Reaction Decoder Tool (RDT): extracting features from chemical reactions. *Bioinformatics* **32,** 2065–2066 (2016).
6. Huß, S., Judd, R. S., Koper, K., Maeda, H. A. & Nikoloski, Z. An automated workflow that generates atom mappings for large-scale metabolic models and its application to Arabidopsis thaliana. *The Plant Journal* **111,** 1486–1500 (2022).
7. Haraldsdóttir, H. S., Cousins, B., Thiele, I., Fleming, R. M. & Vempala, S. CHRR: coordinate hit-and-run with rounding for uniform sampling of constraint-based models. *Bioinformatics* **33,** 1741–1743. ISSN: 1367-4803. eprint: `https://academic.oup.com/bioinformatics/article-pdf/33/11/1741/49039957/bioinformatics\_33\_11\_1741.pdf`. `https://doi.org/10.1093/bioinformatics/btx052` (Jan. 2017).
8. Heirendt, L. *et al.* Creation and analysis of biochemical constraint-based models using the COBRA Toolbox v. 3.0. *Nature protocols* **14,** 639–702 (2019).
9. Lewis, N. E. *et al.* Omic data from evolved E. coli are consistent with computed optimal growth from genome-scale models. *Molecular systems biology* **6,** 390 (2010).
10. Yuan, J., Fowler, W. U., Kimball, E., Lu, W. & Rabinowitz, J. D. Kinetic flux profiling of nitrogen assimilation in Escherichia coli. *Nature chemical biology* **2,** 529–530 (2006).
11. Grankvist, N. *et al.* Global 13C tracing and metabolic flux analysis of intact human liver tissue ex vivo. *Nature Metabolism* **6,** 1963–1975 (2024).
12. Antoniewicz, M. R., Kelleher, J. K. & Stephanopoulos, G. Determination of confidence intervals of metabolic fluxes estimated from stable isotope measurements. *Metabolic engineering* **8,** 324–337 (2006).
13. Long, C. P. & Antoniewicz, M. R. High-resolution 13C metabolic flux analysis. *Nature protocols* **14,** 2856–2877 (2019).
